# Long-term outcome after implantation of a nickel-containing cemented hip stem on the right in a patient with type IV sensitization to nickel sulphate

**DOI:** 10.3205/iprs000144

**Published:** 2019-12-12

**Authors:** Sarah Armonies, Dirk Zajonz, Regina Treudler, Andreas Roth, Mohamed Ghanem

**Affiliations:** 1 Department of Orthopedics, Traumatology and Plastic Surgery, University Hospital Leipzig, Germany; 2 Department of Dermatology, Venerology and Allergology, University Hospital Leipzig, Germany

**Keywords:** nickel-sulfate allergy, joint replacement, type IV sensitization

## Abstract

Type IV allergies to nickel sulfate, potassium dichromate and/or cobalt chloride are supposed to be associated with aseptic loosening, pain or infections in patients with hip arthroplasty. However, there is debate on any causal relation between type IV sensitization to metals and any of these complications. We report on a patient with suspicion of pre-existing type IV sensitization to nickel sulfate and potassium dichromate who did not show any complications after hip arthroplasty.

## Zusammenfassung

Es wird angenommen, dass Typ-IV-Allergien auf Nickelsulfat, Kobaltchlorid und/oder Kaliumdichromat mit einer erhöhten Rate an Komplikationen nach Implantation einer Hüftendototalendoprothese wie Osteolysen, aseptischen Lockerungen, Schmerzen oder Wundinfektionen in Verbindung gebracht werden können. Nicht hinreichend geklärt scheint bis heute ein möglicher kausaler Zusammenhang zwischen diesen Komplikationen und Typ-IV-Sensibilisierungen gegen Metalle.

Wir berichten über einen Fall, bei dem trotz des dringenden Verdachtes auf eine Sensibilisierung auf Nickelsulfat und Kaliumdichromat nach Implantation einer nickelhaltigen Totalendoprothese keine Komplikationen aufgetreten sind.

## Introduction

Patients with type IV allergies to nickel sulphate, potassium dichromate and/or cobalt chloride are supposed to develop aseptic loosening, pain or infections following joint arthroplasty, if the above mentioned materials come in direct contact with bones and tissues.

However, there is debate on any causal relation between type IV sensitization to metals and any of these complications. We report on a patient with suspicion of preexisting type IV sensitization to nickel sulphate and potassium dichromate who did not show any complications after hip arthroplasty with non-titanium covered stem.

## Case description

### Medical history

A 67-year-old man complained of progressive hip pain for 8 years. Activities of daily living, especially walking distance, were limited. Physiotherapy and non-steroidal anti-inflammatory drugs could not bring any relief. Clinical examination of the patient showed a right-sided limping, positive Trendelenburg’s sign and a range of motion of the right hip of: extension/flexion 0/0/90°, abduction/adduction 20/0/10° and external rotation/internal rotation 30/0/20°. Total hip replacement was indicated. 

In addition, the patient suffered from arterial hypertension, benign prostatic hyperplasia, diabetes mellitus type II and latent hypothyroidism. 20 years prior to the scheduled surgery, a patch testing showed a type IV sensitization to a mix of fragrances and propipocainhydrochlorid. Moreover, the patient reported on eczema of the feet after wearing leather shoes. Hence, an allergy to potassium dichromate was suspected. Further, he reported on eczema after wearing nickel-containing materials. This disappeared after he went in retirement. A preoperative patch testing was not performed. 

**Figure 1:**
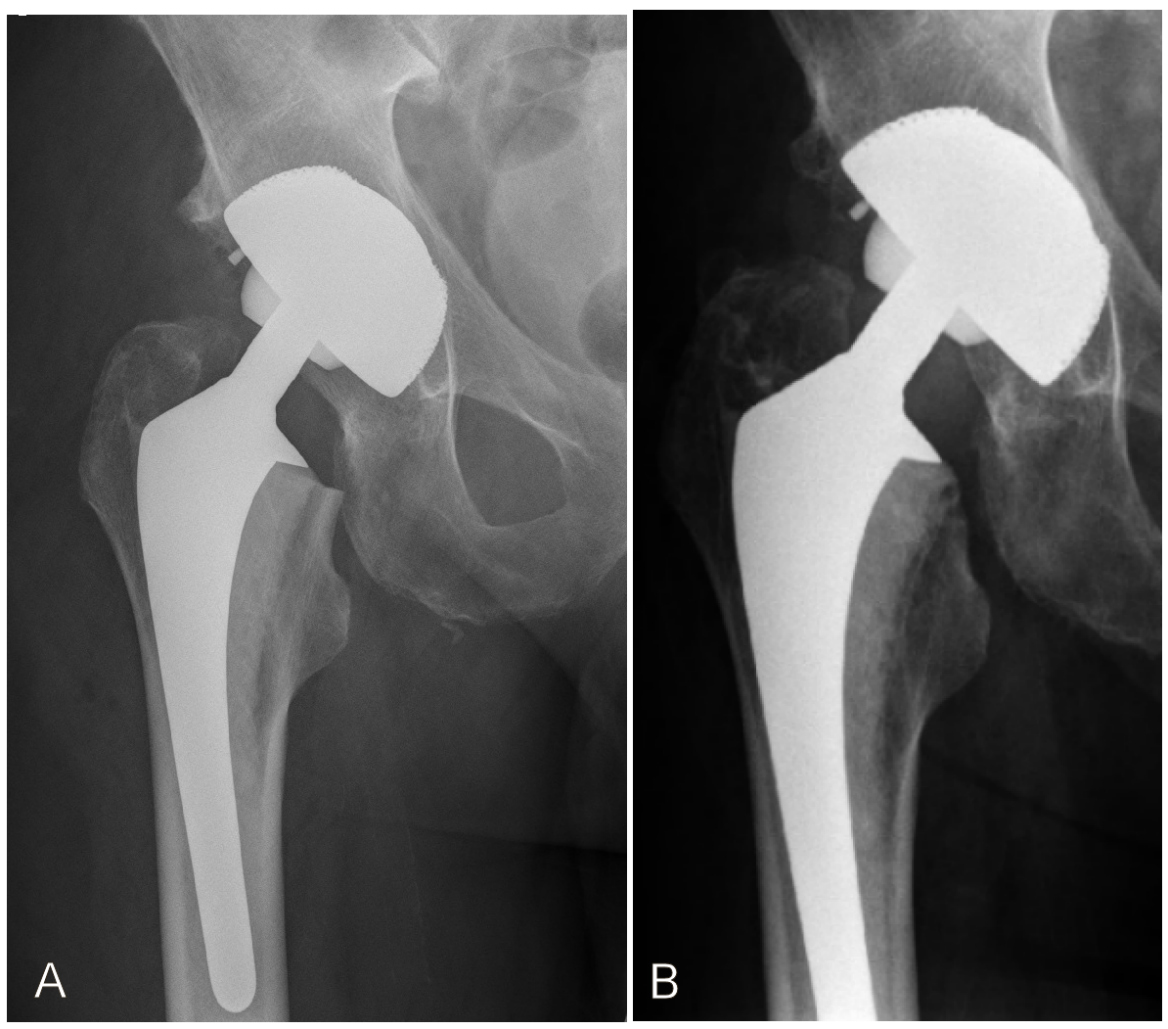
Postoperative X-ray of the right hip 4 days after operation (A) and 8 years later (B). The X-ray 8 years later (B) was done in an external clincic, so unfortunately the complete endosprosthesis is not illustrated. The metaphyseal part shows no loosing. The patient is content and has no symptoms or complaints. Therefore, a current x-ray imaging is unnecessary.

The preoperative planning showed that the second largest cementless titanium-covered femoral stem would be adequate. Yet, intraoperative probation showed that the largest cementless stem was too small. An experienced allergologist was consulted during surgery. There were two options to consider: Girdlestone and second setting implantation of a custom-made or specially ordered large titanium-covered stem or risking implantation of cemented nickel-containing non-titanium covered stem. In this case we favored the second option and planned for postoperative control in the department of allergology. The postoperative course was without any complications (Figure 1A (Fig. 1)). After 3 months, the patient consulted the allergologist. To prevent iatrogenic sensitization, a patch testing of nickel-sulphate and potassium-dichromate was deliberately avoided. Clinical examination was sufficient and it showed no pathological findings.

### Follow-up 

Eight years after implantation of nickel-containing cemented femoral stem, the current clinical examination shows no general or local pathological signs (Figure 1B (Fig. 1)). The patient is content and does not report any general or orthopedic complaints. Walking aids are not used, painkillers are not taken and the walking distance is unlimited. The right hip shows a free range of motion: extension/flexion 0/0/90°, abduction/adduction 40/0/30°, external rotation/internal rotation 45/0/30°. Further, clinical examination shows equal leg length, no Trendelenburg’s sign and intact peripheral circulation and neurological findings. 

The Harris Hip Score (HHS) was 79 points, the Western Ontario and McMaster Universities Osteoarthritis Index (WOMAC) score was 37 points, which is considered very good.

Further, the patient reports no contact dermatitis since the right-sided total hip replacement. Clinical examination showed no evidence of any pathological skin changes.

## Discussion

In recent years and decades, the worldwide number of joint replacement surgery has increased. In 2016, the number of total hip replacement in Germany amounted to 137,295 [1]. In light of this, discussions about the effects of possible contact allergies on the hip joint endoprosthetic components are increasingly becoming the focus of experts. 

Allergic reactions manifest themselves as cutaneous and extracutaneous symptoms [2]. For example, type IV sensitizations to nickel sulphate, potassium dichromate and cobalt chloride are associated with an increased risk of aseptic loosening, persistent pain, and peri-implant osteolysis in endoprosthesis [3], [4]. In particular, materials such as nickel sulphate, potassium dichromate and cobalt chloride, which constitute most of the alloys of a hip joint endoprosthesis, have been previously detected as allergens [5], [6], [7].

It is known that type IV sensitizations can lead to activation of T-lymphocytes, which clinically promote complications such as osteolysis, endoprosthetic loosening and, as a result, the need for revision surgery [7]. Magone et al. describe in their review that osteolysis can be caused by released metal ions, which can occur especially in abrasion and metal-metal pairings. As a result, proinflammatory cytokines are activated and an inflammatory cascade is triggered, which can lead to osteolysis [8]. However, such complications are less likely, if further investigations are carried out prior to surgery [4].

In our reported case, patient history makes us suspect sensitization to nickel sulphate and potassium dichromate, which are standard components of hip endoprosthetic devices [5]. In this context, it should be pointed out that supposed nickel allergies/sensitizations are mostly based on information obtained from patients based on previous allergic reaction to nickel sulphate. This is a common problem that Schnuch et al. already described in 2002. They noted that the 15% prevalence of contact sensitization to nickel sulphate in their cohort was limited to the totality as many patients reported to have a nickel allergy, which was not confirmed by well-established dermatological tests. This would mean that there is a significant number of unreported cases [9]. Accordingly, the Europe-wide prevalence of nickel allergies is reported to be 9–18% [9], [10], [11], [12]. According to such information, it can be assumed that the possible consequences of contact allergies in the case of hip arthroplasty affect every 10^th^ inhabitant and are therefore quite relevant in this aspect. On the other hand, the clinical relevance has to be analyzed. Individual studies point to the fact that, despite high rates of sensitization to nickel sulphate and potassium dichromate, only a few patients have clinical manifestations. Accordingly, dermatological efflorescence such as eczema would be more in the foreground, as peri-implant infections and loosening of the implants [13], [14], [15], [16]. 

Thus, neither postoperative eczema, nor a loosening or infection could be detected upon follow-up clinical examination. Rather, the X-ray examination performed in the previous year showed no signs of loosening. Likewise, neither osteolysis, nor increased pain or infection were identified [3], [7], [8]. Further, low grade infection and arthrofibrosis should be considered, especially in cases of suspected metal allergy [17]. Thus, Thomsen et al. stated that this should be diagnosed by arthroscopically drawn samples [18], [19]. In our case, there weren’t any general or local pathological manifestations. According to HHS and WOMAC score the outcome was very good. This result is consistent with scientific findings such as those of Thyssen et al., which compare revision rates of hip endoprosthesis in the presence of metal allergy to the results of a control group. They showed that the prevalence of revisions was the same in both groups and that no increased complications occurred in the presence of metal allergy [20]. In this context, it should be noted that in our presented case a cemented stem was implanted. So, the cement sheath possibly prevented direct contact of metal with the surrounding bone and soft tissues. Nevertheless, it should be noted that the bone cement and its constituents can cause type IV sensitizations [21]. As reported in literature, a direct contact of implants to the skin could be a trigger of cutaneous symptoms e.g. dermatitis [2], [22]. However, it should be kept in mind that possible skin reactions to metals are not one to one to transfer to the synovium. Thomsen et al. reported in their review that a transferability of skin reaction to metals on the synovial membrane was questionable [18], [19].

In the overall evaluation of our presented case, it should be noted that despite the urgent clinical suspicion of nickel sulphate and potassium dichromate sensitization, no complications occurred after implantation of a hip joint endoprosthesis containing these materials.

## Conclusions

The presented case indicates that existing sensitizations to nickel sulphate and potassium dichromate are not necessarily associated with increased complications after total hip replacement. Preoperative epicutaneous testing in the case of a known metal allergy or sensitization is critically discussed in literature [4], [23], [24]. To obtain a valid statement, further methodically designed studies should be performed. 

## Notes

### Informed consent

The patient has agreed on publication. A written consent is documented.

### Competing interests

The authors declare that they have no competing interests.
